# Ziprasidone as Adjunctive Therapy in Severe Bipolar Patients Treated with Clozapine

**DOI:** 10.1155/2014/904829

**Published:** 2014-04-07

**Authors:** Natalia Bartolommei, Francesco Casamassima, Laura Pensabene, Federica Luchini, Antonella Benvenuti, Antonello Di Paolo, Luca Cosentino, Mauro Mauri, Lorenzo Lattanzi

**Affiliations:** Dipartimento di Medicina Clinica e Sperimentale, Sezione di Psichiatria, Università di Pisa, Azienda Ospedaliero-Universitaria Pisana, Via Roma, No. 67, 56126 Pisa, Italy

## Abstract

*Aim*. To confirm the efficacy and tolerability of ziprasidone as adjunctive therapy in bipolar patients partially responding to clozapine or with persisting negative symptoms, overweight, or with metabolic syndrome. *Methods*. Eight patients with psychotic bipolar disorder were tested with the BPRS, the HAM-D, and the CGI at T0 and retested after 2 weeks (T1). Plasma clozapine and norclozapine levels and BMI were tested at T0 and T1. *Results*. Ziprasidone was well tolerated by all the patients. BPRS and HAM-D scores were reduced in all patients. BMI was reduced in patients with a BMI at T0 higher than 25. Plasma levels of clozapine and norclozapine showed an irregular course.

## 1. Introduction

Atypical antipsychotics (also known as second generation antipsychotics, SGAs) have proven effective in the treatment of schizophrenia and schizoaffective disorder yielding improvements in both positive and negative symptoms [1–11].

Many SGAs are also indicated for the treatment of the different phases of bipolar disorder (BD) (i.e. acute mania and depression and maintenance) in monotherapy and in association with mood stabilizers. The SGAs vary widely in their efficacy/tolerability profiles allowing a better personalization of pharmacotherapy [9, 12–15].

SGAs have represented a meaningful improvement in clinical management of BD but a substantial percentage of patients keep on experiencing relapses, continuous cycling, persistent affective and psychotic symptoms, and functional deterioration [14, 16, 17].

Clozapine, the “gold standard” of SGAs, has never been approved for the treatment of bipolar disorder, though it has showed efficacy in acute mania [18–22] and in the treatment of severe psychotic bipolar disorder. This latter has been suggested to represent a possible intermediate clinical phenotype placed in the continuum of major psychoses bridging typical bipolar disorder to schizophrenia through schizoaffective disorder [9, 20, 21, 23–28].

A significant percentage of patients with severe psychotic bipolar disorder exhibit poor or incomplete response to usual monotherapy and combination treatments of adequate dosages and duration. Clozapine has been proposed as a suitable rescue choice to manage such complex conditions including uncontrollable mood instability, agitation, insomnia, psychosis, and aggressiveness. Unfortunately, even the addition of clozapine to the therapeutic strategy often leads only to a partial response [29, 30]. Moreover, clozapine treatment is burdened with several short- and longer-term side effects such as sialorrhoea, oversedation, increased appetite leading to weight gain, new-onset diabetes, dyslipidemia and metabolic syndrome [31].

Many studies focusing mostly on schizophrenia samples have proposed distinct adjunctive antipsychotics in order to promote clinical remission, to attempt a clozapine dose reduction, and/or to mitigate its side effects [27, 32–46].

Although the combination of two or more antipsychotics in bipolar disorder is poorly documented and, to our knowledge, not supported by randomized clinical trials, it is a common strategy in clinical practice [47]: about 15% of psychotic bipolar outpatients and 50% of inpatients receive more than one antipsychotic [48–50].

We have previously reported that the augmentation of clozapine with aripiprazole may be safe and effective in severe psychotic schizoaffective and bipolar disorder which failed to respond to SGAs [51].

Ziprasidone is an atypical antipsychotic recently introduced in the Italian market, approved for the treatment of schizophrenia in adults and also for the treatment of bipolar manic or mixed episodes of moderate severity in adults, adolescents, and children (aged between 10 and 17), with a reported low potential for extrapyramidal side effects.

Given its antagonistic activity on serotonin 5-HT2A, 5-HT1D, and 5-HT2C receptors and the agonist partial activity on 5-HT1A receptors as well as the moderate* in vitro*-inhibitory activity on serotonin and noradrenaline transporters, ziprasidone has been suggested to display efficacy on depressive and negative symptoms [42, 52, 53]. Ziprasidone, in conjunction with mood stabilizers, could promisingly offer additional mood regulating, antidepressant, and anxiolytic actions [43, 54, 55].

Its low affinity for H1 histaminergic, alpha1 adrenergic, and M1 muscarinic receptors predicts a safe tolerability profile with a low potential, respectively, for weight gain, hyperglycemia, cholesterol and triglyceride dysregulation, orthostatic hypotension and cardiovascular side effects, sedation, and cognitive impairment [43, 56]. Because of this favourable side effect profile and low risk of pharmacologic interactions, ziprasidone is a suitable candidate for clozapine augmentation.

The association of ziprasidone to an ongoing clozapine treatment has proven effective in severe psychotic patients bringing about improvements of negative/cognitive symptoms and of clozapine related side effects, with only a minor QTc interval prolongation [11, 37, 44, 57, 58].

Aims of this paper are to report on the potential efficacy and tolerability of the clozapine-ziprasidone association in a case series of severe psychotic bipolar patients resistant to previous medication trials and to provide preliminary data on possible pharmacokinetic interactions between the two antipsychotics.

## 2. Material and Methods

Patients were recruited in the Inpatient Psychiatric Unit of the Department of Psychiatry at Pisa University. All patients received a diagnosis of bipolar disorder with psychotic symptoms by the treating clinician according to the DSM-IV criteria [59].

*Eligibility criteria* were the following: (1) age of 18–65 years, (2) resistance/inadequate response to at least two trials of mood stabilizers (i.e. lithium salts, valproic acid, and carbamazepine) and/or of antipsychotics prescribed at adequate dosages, for at least six months, (3) treatment with clozapine at least six months ago, (4) relevant mood swings associated with persistent psychotic symptoms despite the treatment with clozapine, (5) no contraindications to ziprasidone treatment (e.g., prolonged QTc interval), (6) normal bone marrow and hepatic and renal functions, (7) capability to attend follow-up visits, and (8) proved compliance to treatment as indicated by blood levels monitoring.

On the contrary,* exclusion criteria* were as follows: (1) history of drug abuse/dependence within six months from enrolment, (2) diagnosis of neurologic disorders such as Parkinson's disease, epilepsy, and myasthenia gravis, (3) jaundice or haematological diseases, (4) pregnancy or breast-feeding, and (5) current major depression.

Administration of other psychotropic medications other than clozapine and ziprasidone was allowed as clinically needed and recorded as well.

According to usual monitoring protocols implemented at our Inpatient Psychiatric Unit, patients underwent assessments with a number of clinical rating scales, systematic recording of side effects, laboratory tests, body mass index (BMI) monitoring, and measurement of clozapine and norclozapine plasma levels.

Clozapine and norclozapine plasma levels recorded within patients' case report forms were considered for the aims of the present study on the day before (T0) and 15 days after (T1) ziprasidone introduction. The measurement of plasma drug levels was performed in fasting condition three hours after the clozapine morning dose, which corresponds approximately to the time of clozapine peak concentration. BMI and concomitant therapies information was collected at T0 and T1 as well as any unwanted effects of medications. All patients provided written consent to the review of their case histories.

### 2.1. Efficacy and Tolerability Assessments

The Mini International Neuropsychiatric Interview (M.I.N.I.) was administered at T0 to confirm diagnosis. Clinical features and disease severity were evaluated by means of the Brief Psychiatric Rating Scale 24 items (BPRS), the Hamilton Depressive Rating Scale (HAM-D), and the Clinical Global Improvement (CGI).

### 2.2. Laboratory Analysis

Blood samples (5 mL) for clozapine plasma levels measurement were withdrawn from a peripheral vein of the forearm, collected within heparinized tubes, and immediately centrifuged. Plasma was stored at −20°C until the actual measurement of drug levels.

Plasma concentrations of clozapine and its active metabolite were determined by the high performance liquid chromatography (HPLC) method, using a commercially available kit (Chromsystems, Munchen, Germany), following the instructions of manufacturers. The clozapine/norclozapine plasma concentration ratio was calculated as an index of drug metabolism in patients, while levels of the drug and the metabolite were normalized by the daily clozapine dose in order to reduce interpatient variability.

### 2.3. Statistical Analysis

BPRS, HAM-D, and CGI scores before and after ziprasidone association were compared using the Wilcoxon signed rank test. Statistical significance was set at 2-sided *P* < 0.05 (StataCorp. 2007. Stata Statistical Software: Release 10. College Station, TX: StataCorp LP).

## 3. Results

### 3.1. Clinical Characteristics

Eight patients (6 males and 2 females) were enrolled from May 2011 to September 2011. All subjects were diagnosed with bipolar disorder, mixed episode with psychotic features. One patient received the additional diagnosis of social phobia and one patient of comorbid panic disorder.

Before ziprasidone augmentation, patients had been assuming clozapine at a mean dosage of 140.6 mg daily (range 50–250 mg daily) for at least 6 months. All patients were at clozapine steady state and previous controls had demonstrated stable plasma concentrations. Moreover, all patients had been taking all the other drugs (see Table 3) for at least a period of 30 days before the study initiation.

Ziprasidone was added to the ongoing therapy starting at a dose of 40 mg twice daily up to a final total daily dose of 120–160 mg/day (mean dose: 155 mg/day).

### 3.2. Metabolic Parameters

Cholesterol and triglycerides plasma levels were tested at T0. No patients showed severe metabolic dysfunctions (see Table 1). One patient had a second grade obesity (BMI 36.6), two patients had a first grade obesity (BMI of 30 and 32, resp.), three were overweight (BMI of 27.7, 27, and 26, resp.), and two had a normal BMI (see Table 4).

### 3.3. Clozapine, Norclozapine Plasma Levels and Clozapine/Norclozapine Ratio

Mean plasma levels of clozapine and norclozapine at T0 were 0.172 mg/L and 0.143 mg/L, respectively. Mean plasma levels of clozapine and norclozapine at T1 were 0.304 mg/L and 0.119 mg/L, respectively.

After the adjunction of ziprasidone the clozapine dosage was reduced in 2 patients and increased in other 3 patients. As a whole, plasma levels of clozapine and norclozapine fluctuated for each patient irregularly and irrespective of drug dosage modifications.

### 3.4. Concomitant Treatments

After the augmentation with ziprasidone concomitant psychotropic treatments were reduced in all patients with the exception of one (see Table 2).

### 3.5. Severity Assessment

At T1, the majority of the patients displayed a significant improvement of symptom severity as assessed by BPRS (*z* = 2.52, *P* = 0.01), CGI (*z* = 2.64, *P* < 0.01), and HAM-D (*z* = 2.18, *P* = 0.03) rating scales. Specifically, the mean BPRS* anxiety depression domain* score decreased from 11.4 at T0 to 8.1 at T1; the* anergy domain* varied from 12.9 to 8.7;* the thought disorder domain* decreased from 13.4 to 9.5; the* psychomotricity domain* changed from 5.9 to 4.75; lastly, an improvement from 7.7 at T0 to 5.7 at T1 was detected in the* suspiciousness hostility domain.*

One patient showed an HAM-D score worsening at T1 due to a higher level of psychic and somatic anxiety. This patient was the one diagnosed with comorbid panic disorder (Table 3).

### 3.6. Side Effects

No treatment emergent side effects were apparently attributable to ziprasidone prescription.

## 4. Supplementary Data

The reduction of valproic acid was possible in one patient (number 8). Two patients needed to stop lithium therapy because of the onset of psoriasis and the worsening of glomerulonephritis, respectively. In patients 2, 3, 5, and 6 haloperidol was suspended. In patient 2 escitalopram was withdrawn as well. Finally, bromperidol was stopped in patient 7.

## 5. Discussion

The efficacy and safety of the combination ziprasidone- clozapine in patients with schizophrenia have been proved in previous studies [11, 37, 44, 57, 58]. The efficacy of ziprasidone as monotherapy or in combination with mood stabilizers in patients with bipolar I disorder has been previously reported [60, 61]. Conversely, other authors failed to show efficacy of ziprasidone for different bipolar phases [54, 62].

To our knowledge, this is the first study to evaluate efficacy and tolerability of the clozapine-ziprasidone combination for persistent mood instability and psychotic symptoms in bipolar patients with burdensome overweight and other treatment emergent side effects.

The augmentation of clozapine with ziprasidone was associated with unequivocal improvements of both psychotic and affective symptoms. In fact, all of the patients showed a score reduction on different BPRS domains and amelioration of depressive symptoms as assessed by the HAM-D. Our findings confirm previous reports suggesting a possible antidepressant effect of ziprasidone in patients suffering from bipolar type I or type II depression [55, 63]. Furthermore, we noticed that the addition of ziprasidone alleviated the clinical burden of clozapine related side effects such as sialorrhea and weight gain.

In our sample, only one patient with comorbid panic disorder worsened after the addition of ziprasidone because of an increase of the HAM-D anxiety score. This may suggest caution or a different dosage titration of ziprasidone in subjects with anxiety symptoms, slightly in contrast with prior open-label and placebo-controlled evidences of ziprasidone efficacy in refractory generalized anxiety disorder (GAD) [64, 65]. However, in the double-blind controlled study on GAD, ziprasidone was prescribed to lower flexible doses (i.e., 20–80 mg versus 80–160 daily in our report).

Kaushik et al. [66] described the case of a patient aged 43 with schizoaffective disorder who had showed psychomotor activation after the prescription of high dose ziprasidone treatment. After nine days of ziprasidone monotherapy (at a dose of 320 mg/day), the authors reported the appearance of psychomotor restlessness, irritability, anxiety, and akathisia [66]. According to the data available in the literature and based on what we observed in our sample, as well as in relation to the pharmacodynamics of ziprasidone, it could be argued that lower doses are associated with more anxiolytic properties and fewer extrapyramidal side effects while higher doses may be associated with akathisia and the loss of anxiolytic effects.

As shown by the nonlinear variations of clozapine/norclozapine plasma levels before and after ziprasidone introduction, the efficacy of ziprasidone was not related to a pharmacokinetic interaction with clozapine. Accordingly, Ziegenbein et al. (2005) have demonstrated that clozapine and ziprasidone undergo different metabolic pathways, with the former metabolized by the cytochrome P450 system and the latter mostly through aldehyde oxidation [42, 67, 68].

The additional dopamine D2 antagonistic action of ziprasidone may enhance the relatively weak D2 blockade exerted by clozapine and partially explain the clinical improvement produced by this combination. Furthermore, ziprasidone displays serotonin and noradrenaline reuptake inhibition properties and 5HT1C agonist activity [67]; this is offering other reasonable explanations for its benefits on mood and psychomotor symptomatology.

Ziprasidone was well tolerated in all of the patients. No patient needed to stop the drug due to treatment emergent side effects. Adjunctive ziprasidone resulted in a BMI reduction in the vast majority of subjects, according to other published findings [58, 69], except for those two patients who had a normal BMI at the initial assessment. Moreover, Ziprasidone was associated with the withdrawal of concomitant antipsychotics in all the patients but two (see Table 2). These findings are promisingly in keeping with results from schizophrenia trials and small studies suggesting that ziprasidone may allow a dose reduction/discontinuation of coprescribed medications, decrease appetite, and even bring about a sizeable weight loss [57, 58, 69].

## 6. Study Limitations

This is a small naturalistic short-term case series of the clozapine-ziprasidone combination for severe psychotic bipolar patients. The promising results we have shown both in improved efficacy and in tolerability need to be confirmed in larger size and longer-term studies focused also on relapse prevention and assessment of global functioning. Given that clozapine is not indicated for bipolar disorder and should be considered an exceptional rescue treatment, it is unlikely that controlled trials will be performed. Therefore, our choices will have to rely mostly on clinical experience and case reports. In future, a more precise evaluation of psychopathological dimensions (e.g. energy levels, mood, proneness to psychosis, sleep, etc.) affected by ziprasidone augmentation will help to refine our opinion on this therapeutic strategy. In the absence of other risk factors for QTc prolongation/cardiac arrhythmias, ziprasidone prescription does not demand electrocardiogram monitoring [70]. Nevertheless, it would be interesting to add information about QTc variations in next studies, as Kuwilsky et al. (2010) have already recorded a statistically significant QTc elongation in schizophrenic patients treated with the clozapine-ziprasidone association [57].

## Figures and Tables

**Table 1:** Metabolic parameters before and after the addition of ziprasidone.

Patients	Cholesterol levels mg/dL T0	Cholesterol levels mg/dL T1	LDL mg/dL T0	LDL mg/dL T1	HDL mg/dL T0	HDL mg/dL T1	Triglycerides mg/dL T0	Triglycerides mg/dL T1	BMI T0	BMI T1
1	174	—	112	—	41	—	78	—	36.2	33.9
2	168	108	111	60	28	20	211	166	32.2	31.4
3	147	—	85	—	41	—	144	—	27	26.6
4	113	—	49	—	30	—	187	—	27.7	27.7
5	218	184	139	126	43	36	183	112	30	29.1
6	117	—	62	—	—	—	184	—	24.5	25.1
7	189	—	109	—	53	—	113	—	26.2	25.7
8	165	—	105	—	37	—	84	—	23.6	24.3

**Table 2:** Concomitant treatments at T0 and T1.

Patients	Concomitant treatments at T0	Concomitant treatments at T1
1	Haloperidol 9 mg Chlorpromazine 50 mg Chlordemethyldiazepam 1.8 mg Bisoprolol 10 mg	Haloperidol 9 mg Chlorpromazine 50 mg Chlordemethyldiazepam 1.8 mg Bisoprolol 10 mg

2	Haloperidol 3 mg Escitalopram 10 mg Fat acid 1000 mg Fenofibrate 145 mg	Fat acid 1000 mg Fenofibrate 145 mg

3	Orphenadrine 4 mg Clonazepam 1.5 mg Haloperidol 4 mg Levetiracetam 500 mg	Orphenadrine 4 mg Clonazepam 1.5 mg Levetiracetam 500 mg

4	Haloperidol 1.2 mg Valproic acid 600 mg Lithium carbonate 900 mg Orphenadrine 4 mg	Haloperidol 1.2 mg Valproic acid 600 mg Orphenadrine 4 mg

5	Lithium carbonate 600 mg Haloperidol 2.5 mg Citalopram 20 mg Levotiroxin 50 mcg Allopurinol 150 mg Clonazepam 1 mg	Lithium carbonate 600 mg Levotiroxin 50 mcg Citalopram 20 mg

6	Haloperidol 3 mg Lithium carbonate 300 mg Chlordemethyldiazepam 0.8 mg Ramipril 5 mg Atenolol 50 mg	Chlordemethyldiazepam 0.8 mg Ramipril 5 mg

7	Bromperidol 2.5 mg Lithium Carbonate 300 mg	Lithium Carbonate 300 mg

8	Lithium Carbonate 600 mg Valproic acid 1500 mg	Lithium Carbonate 600 mg Valproic acid 1250 mg ECT

**Table 3:** Percentage of improvement at BPRS, HAM-D, and CGI.

Patients	BPRS	HAM-D	CGI
1	13.54%	16.4%	28.5%
2	10.4%	8.9%	14.2%
3	3.6%	−3%	14.3%
4	11.9%	13.4%	14.2%
5	14.3%	6%	14.3%
6	4%	10.4%	14.2%
7	13.6%	0%	14.3%
8	17.5%	12%	28.6%

**Table 4:** Percentage of BMI improvement after ziprasidone augmentation.

Patients	BMI T0	BMI T1	Percentage of BMI improvement
1	36.2	33.9	↓ 6.35%
2	32.2	31.4	↓ 2.48%
3	27	26.6	↓ 1.48%
4	27.7	27.7	=
5	30	29.1	↓ 3%
6	24.5	25.1	↑ 2.45%
7	26.2	25.7	↓ 1.9%
8	23.6	24.3	↑ 3%
